# Genetic Engineering: A Promising Tool to Engender Physiological, Biochemical, and Molecular Stress Resilience in Green Microalgae

**DOI:** 10.3389/fpls.2016.00400

**Published:** 2016-03-31

**Authors:** Freddy Guihéneuf, Asif Khan, Lam-Son P. Tran

**Affiliations:** ^1^Botany and Plant Science, School of Natural Sciences, Ryan Institute, National University of Ireland GalwayGalway, Ireland; ^2^Research Group Germline Biology, Centre for Organismal Studies (COS), Heidelberg UniversityHeidelberg, Germany; ^3^Plant Abiotic Stress Research Group & Faculty of Applied Sciences, Ton Duc Thang UniversityHo Chi Minh City, Vietnam; ^4^Signaling Pathway Research Unit, RIKEN Center for Sustainable Resource ScienceTsurumi, Japan

**Keywords:** microalgae, abiotic stresses, genetic engineering, strain improvement, potential applications

## Abstract

As we march into the 21st century, the prevailing scenario of depleting energy resources, global warming and ever increasing issues of human health and food security will quadruple. In this context, genetic and metabolic engineering of green microalgae complete the quest toward a continuum of environmentally clean fuel and food production. Evolutionarily related, but unlike land plants, microalgae need nominal land or water, and are best described as unicellular autotrophs using light energy to fix atmospheric carbon dioxide (CO_2_) into algal biomass, mitigating fossil CO_2_ pollution in the process. Remarkably, a feature innate to most microalgae is synthesis and accumulation of lipids (60–65% of dry weight), carbohydrates and secondary metabolites like pigments and vitamins, especially when grown under abiotic stress conditions. Particularly fruitful, such an application of abiotic stress factors such as nitrogen starvation, salinity, heat shock, etc., can be used in a biorefinery concept for production of multiple valuable products. The focus of this mini-review underlies metabolic reorientation practices and tolerance mechanisms as applied to green microalgae under specific stress stimuli for a sustainable pollution-free future. Moreover, we entail current progress on genetic engineering as a promising tool to grasp adaptive processes for improving strains with potential biotechnological interests.

## Introduction

**FIGURE 1:**
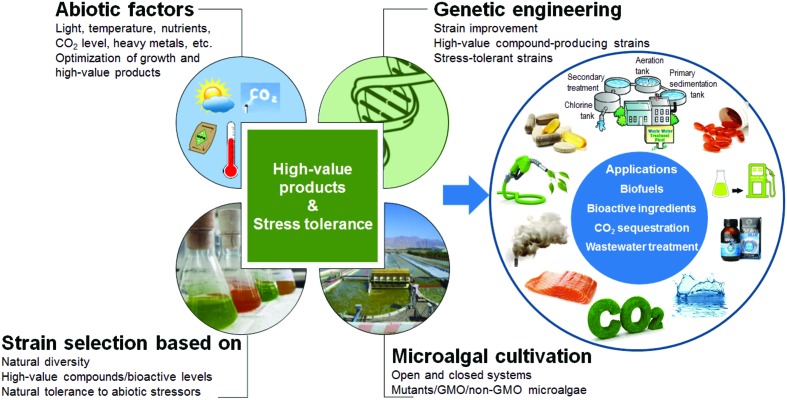
**Illustrated scheme for improving microalgal strains for potential biotechnological interests via genetic engineering.** GMO, genetically modified organism.

Due to their taxonomic and biochemical diversity, microalgae symbolize an unconventional source of molecules (Stengel et al., 2011). For example, the inherent nature of microalgae to accumulate lipids, carbohydrates, and secondary metabolites under abiotic stress can be applied in a biorefinery concept for generation of value-added compounds (Markou and Nerantzis, 2013). Nonetheless, transgenic microalgae impact diverse businesses such as energy, human and animal nutraceuticals, pharmaceuticals, health, beauty, and exquisite chemicals (Rasala et al., 2014). As naturalized for bacteria, yeasts, and plants, genetic engineering, therefore, constitutes promising strategy for studying abiotic stress responses in microalgae through novel phenotypes and specific traits. Conversely, microalgae as model photosynthetic organisms represent an ideal experimental system to study major plant processes, as mimicking algal strategies to sense, respond and cope with abiotic stress can radically improve growth and metabolite productivity in plants. Over the years, significant advances in genetic manipulation of green microalgae have been achieved (Mayfield and Golden, 2015), as exemplified through production of omega-3 fatty acids, carotenoids, biofuels, as well as improved photosynthetic growth (Gimpel et al., 2015). Despite tremendous advances in sequenced genome, in particular for chlorophytes and heterokonts (i.e., diatoms), major obstacles feature need of additional well-established transformation vectors, potential inability to engineer and specifically localize transgenic proteins to sub-cellular locations and robust expression of multiple nuclear-encoded transgenes (Gong et al., 2011; Scranton et al., 2015). In this minireview, we discuss recent examples highlighting futuristic use of genetic engineering to design strains with potential biotechnological interests (**Figure 1**). Moreover, we surmise major abiotic stress resilience strategies envisaged in unicellular eukaryotic green microalgae.

### Current Progress on Genetic and Metabolic Engineering of Green Microalgae

#### What We Know and What Is Available?

The pursuit of stress-tolerant strains has seen an unprecedented surge of algal transgenesis for recombinant proteins, enhanced photosynthesis, and key metabolite pathways, as well as production of precious nutraceuticals, pharmaceuticals, and biofuels (Gangl et al., 2015). Coupled to this, advanced cultivation of novel species with genome-wide omics (genomics, transcriptomics, lipidomics, and metabolomics) has facilitated successful transformation of microalgae (e.g., *Chlamydomonas reinhardtii*, *Phaeodactylum tricornutum*, *Thalassiosira pseudonana, Porphyridium sp.*, *Nannochloropsis gaditana*, *Ostreococcus tauri*, *Haematococcus pluvialis*, etc.). Previously, lack of genome and transcriptome data, as well as efficient tools for algal transformation prevented their genetic manipulation. Consequently, algal transformation systems had to be revisited, developed anew, including methods to introduce transgenic DNA, identification of suitable promoters, distinct selectable markers and expression vectors, as well as needs for codon usage optimization (Qin et al., 2012; Hlavova et al., 2015).

Despite gigantic successes being achieved, only a few algal species still show firm and stable expression of foreign proteins. Increasing evidence suggests intrinsic algal gene silencing mechanisms; albeit repressive histone H3 lysine methylation, DNA cytosine methylation, RNA interference and microRNA-mediated gene regulation for instable foreign transcript or protein degradation (Kim et al., 2015). Likewise, the knowledge of such transgene evading mechanisms will be useful in regulating efficient protein production in metabolically engineered microalgae. Additionally, the emergence of fast and reliable next-generation sequencing techniques have sped up genome sequencing programs and transcriptomic research (Morozova and Marra, 2008). As a result, it is now possible to directly correlate the ensuing transcriptomic, proteomic, and metabolomic profile with respect to pre-determined environmental stimuli, bridging the gap between microalgal genotype and observed phenotype (Dong and Chen, 2013). In future, a combination of transcriptomic, proteomic, and metabolomic studies will be crucial in demarcating gateways to metabolic flux for boosting algal strain-engineering strategies (Jones and Mayfield, 2012). In addition, a battery of bioinformatic softwares like Algal Functional Annotation Tool, GreenCut2, AlgaGEM, Bag2D, PredAlgo (de Oliveira Dal’Molin et al., 2011; Karpowicz et al., 2011; Lopez et al., 2011; Tardif et al., 2012; Yao et al., 2015), and *in silico* kinetic and metabolic reconstruction models^1^ are now available for gene/protein characterization, gene metabolic alterations, and predicting proteins subcellular localizations at a whole genome level in various algal species. For example, an *in silico* metabolic reconstruction map allowed characterization of light-induced metabolic response for >1000 genes in *Chlamydomonas* (Chang et al., 2011). Likewise, the *Chlamydomonas* resource center^2^ harbors a collection of ~300 plasmids and >2700 strains for sustaining research within diverse aspects of algal cell biology. Additionally, a genome-wide *Chlamydomonas* insertional mutant library^3^ containing >20,000 mutants covering 10,301 genes (58% of the genome) and cataloged at *Chlamydomonas* resource center has been recently reported (Li et al., 2016).

#### Genetic Engineering: Progress and Future Prospects

With an eye toward the future, methods to engineer entire biochemical pathways or multigene traits via homologous/heterologous recombination and development of optimized cloning vectors are underway, as demonstrated by stable integration and subcellular localization of reporter proteins in the chloroplast and nuclear genomes of *C. reinhardtii* (Noor-Mohammadi et al., 2012; Rasala et al., 2014; Scranton et al., 2016). Such a strategy can be advantageous for foreign gene integration, omitting positional side effects and transcriptional/post-transcriptional gene silencing processes associated with nuclear expression (Kim et al., 2015). In addition, successful yet simple nuclear gene targeting systems have been described in *Chlamydomonas* (Zorin et al., 2009). Novel permutations of promoter and 5′UTR, like 16S rRNA promoter and *atpA* 5′UTR have also been illustrated for sufficient heterologous transgene expression (Rasala et al., 2011) and robust expression of transgenic proteins within the *C. reinhardtii* chloroplast (Tissot-Lecuelle et al., 2014). Examples of metabolic engineering in microalgae comprise codon optimized expression of genes *hemH* and *lba* in the chloroplast of *C. reinhardtii* for optimum bio-hydrogen generation (Wu et al., 2011). Recently, synthetic promotors were generated to drive robust nuclear gene expression in *Chlamydomonas* (Scranton et al., 2016).

In search of optimal methods for exogenous DNA delivery within rigid algal cell walls, a zinc oxide nanowire array microdevice system was recently elaborated for *C. reinhardtii* (Bae et al., 2015) with recorded efficiency 6.52 × 10^4^- and 9.66 × 10^4^-fold higher than traditional glass bead beating and electroporation methods. Besides, a biocontainment method for transplastomic microalgae was reported via codon reassignment in the chloroplast of *C. reinhardtii* (Young and Purton, 2015). Such a codon reassignment strategy can be potentially useful for solving imminent problems in chloroplast engineering of genes whose products are toxic to *Escherichia coli* cells. Similarly, a self-cloning-based (cloning DNA from a donor into a recipient, between which the natural exchange of DNA is possible) positive selection system for breeding and genetic transformation of *Pseudochoricystis ellipsoidea* was developed (Kasai et al., 2015). Such recombinant microalgae are considered natural (non-GMO) in Japan and are not governed under the Cartagena domestic law with regards to GMO biosafety (Lusser et al., 2012; USDA Japan Reports, 2014). Such algal molecular breeding techniques may form basis for future large-scale cultivation in outdoor open pond cultivation (Ho et al., 2014).

Finally, reports for high level targeted genome editing in *C. reinhardtii* via zinc finger nucleases (Sizova et al., 2013), transcription activator-like effectors (TALE; Gao et al., 2014), and lately clustered regularly interspersed short palindromic repeat (CRISPR) system are emerging (Jiang et al., 2014). CRISPR-associated protein 9 (CAS9) is an RNA-guided DNA nuclease successfully practiced for targeted mutations into eukaryotic genomes (Dominguez et al., 2016). Although stable transformants of *C. reinhardtii* expressing the CAS9 protein could not be recovered, in future, such studies would serve as gold standard for dedicated nuclear, chloroplast, and mitochondria genome-editing strategies.

### Microalgal Stress Response Strategies

#### Abiotic Stress Tolerance in Green Microalgae

Owing to incredible metabolic plasticity of microalgae (Stengel et al., 2011), numerous studies have outlined ramifications of abiotic stress influencing algal biology and metabolism. High light, temperature, salinity, metals, CO_2_ levels, and nutrient depletion affect growth, photosynthesis, and biochemical composition in a species-specific manner (Perales-Vela et al., 2006; Solovchenko and Khozin-Goldberg, 2013).

One of the most detrimental abiotic stressor damaging algae is high light intensity, when absorption of light energy surpasses the capacity for light utilization in photosynthesis. Several mechanisms termed as non-photochemical quenching (NPQ) exist, to avoid this photodamage (Allorent et al., 2013; Mayank et al., 2015). NPQ consists of three components: qE (energy-dependent quenching), qI (photoinhibitory quenching), and qT (light-state transition; Eberhard et al., 2008; Depauw et al., 2012). The qE increases the thermal dissipation of excessive light energy and represents the major photo-protective process characterizing vascular plants, green microalgae and diatoms (Müller et al., 2001; Roháček et al., 2014). In model green alga *C. reinhardtii*, NPQ is stimulated by variations in pH in the thylakoid lumen, in response to high light intensity, triggering the xanthophyll cycle (XC) and activating the PSII LHC stress-related (LhcSR) proteins (Finazzi et al., 2006; Maruyama et al., 2014; Wobbe et al., 2016). The latter initiates an onset of NPQ by perceiving lumenal pH environment and in a concentration-dependent manner aggravates fluorescence quenching; the XC plays key role in boosting light harvesting and photoprotection (Goss and Jakob, 2010). Nevertheless, studies of their NPQ and XC activities have suggested species-specific differences in process of excessive energy dissipation between six green microalgae (Quaas et al., 2015). Of interest, microalgal species like *Dunalliela* spp. and *H. pluvialis* display the capacity to synthetize and accumulate tremendous amounts of secondary carotenoids (β-carotene and astaxanthin, respectively) under stress (Lamers et al., 2012; Scibilia et al., 2015). In photosynthetic organisms, response to high light involves enormous changes in expression of light-regulated genes and their regulatory components (Lemoine and Schoefs, 2010). Over the years, dedicated gene expression studies with microalgae exposed to different light regimes were undertaken, unveiling peculiar molecular actors associated with light stress responses (e.g., *Lhcx* gene group in diatoms, known to be closely related to *LhcSR* genes of *C. reinhardtii*; Maruyama et al., 2014).

Microalgae further picture a suite of adaptive mechanisms in response to changes in salinity, i.e., passive “osmometer” behavior. Such a profile largely depends on physical and chemical characteristics of the cell wall membrane, regulation of water fluxes, mechanisms to equilibrate intracellular ionic levels using ion-selective channels and carriers, and synthesis or degradation of low-molecular-weight organic solutes (Kirst, 1990). The halotolerant green alga, *Dunaliella salina*, has been described as a model alga for deducing response of plant cells to salt stress (Cowan et al., 1992). Employing a proteomic approach for salinity stress induced proteins in *Dunaliella*, Liska et al. (2004) decoded up-regulation of major Calvin cycle enzymes, starch mobilization enzymes and enzymes involved in redox energy generation; factors involved in protein production and degradation; and an analog of bacterial Na^++^-redox transporters. The results are suggestive of *Dunaliella’s* response to high salinity by enhancing photosynthetic CO_2_ consumption and allocating carbon and energy budget for synthesis of glycerol, its osmo-protective component. On the same lines, salt-stress up-regulated the carotenoid ketolase (*BKT*) in *Chlorella zogingiensis* and enhanced the accumulation of canthaxanthin and astaxanthin. High salinity also stimulated the generation of reactive oxygen species, which in turn triggered the up-regulation of distinct carotenogenic genes and increased build-up of antioxidant carotenoids (Li et al., 2009).

Changes in temperature are known to strongly affect growth, photosynthetic activity and biochemical composition in microalgae (Geider, 1987; Renaud et al., 2002; Claquin et al., 2008). Indeed, higher temperatures often result in decline in protein production and rise in lipid and carbohydrates levels. An increase in polyunsaturated fatty acids (PUFAs) might be one of the ways microalgae adjust to low-temperature and preserve membrane fluidity (Jiang and Gao, 2004). Interestingly, few microalgae exhibit high temperature tolerance up to 40–42°C (Hanagata et al., 1992; Sakai et al., 1995; de-Bashan et al., 2008). Heat shock proteins (HSPs) are molecular chaperones, crucial during heat shock response and ensuing adaptive homeostases against environmental stresses. 17 small HSPs has been identified from complete genome sequences of five diverse algae: *C. reinhardtii*, *Cyanidioschyzon merolae*, *Ostreococcus lucimarinus*, *O. tauri*, and *T. pseudonana* (Waters and Rioflorido, 2007). However, sequence analysis reveals that the diversity and abundance of algal small HSPs does not fully correlate with acclimation to extreme temperatures. Recently, two small *HSP*s have been shown to be up-regulated and activated at a threshold temperature of ~36°C in *C. reinhardtii* (Kobayashi et al., 2014). These results stress the advantage of an exclusive temperature-sensing system in the evolution and resilience to elevated temperatures among microalgae.

#### Mechanisms of CO_2_ Concentration and Heavy Metal Tolerance

Rise in atmospheric O_2_ and decrease in oceanic, dissolved inorganic carbon during eco-genesis enabled photoautotrophic organisms to formulate sophisticated CO_2_-concentrating mechanisms (CCMs). Such mechanisms enhance CO_2_ uptake by increasing CO_2_ concentration around ribulose-1,5-bisphosphate carboxylase/oxygenase (Rubisco), an enzyme of the chloroplast stroma that catalyzes the entry of CO_2_ into the Calvin–Benson–Bassham cycle to maintain sufficient photosynthesis (Raven, 2010). To date, numerous published reports have described low-CO_2_-acclimation/adaptation within some cyanobacteria and unicellular eukaryotes; but, information regarding high-CO_2_ tolerance processes remains elusive. One CO_2_ mitigation strategy consists of selecting and developing strains that stand rigorously high CO_2_ levels to couple microalgal-mediated CO_2_ fixation and production of biofuels and other secondary metabolites (Bhola et al., 2014). Since CO_2_ tolerance varies greatly between species, microalgae have been arbitrarily divided into CO_2_-sensitive (inhibited by <2–5% CO_2_) and CO_2_-tolerant (surviving up to 20% CO_2_) groups (Miyachi et al., 2003). An example of such CO_2_-tolerant species includes chlorophytes *Chlorella* sp. KR-1 and *Chlorococcum littorale* that demonstrate rapid growth at 40 and 60% CO_2_, respectively (Iwasaki et al., 1996; Sung et al., 1999). Principal factors responsible for high CO_2_ tolerance in such extremophile symbiotic species constitute state transitions in photosynthetic apparatus that augment ATP generation, enhanced activity of H^+^-ATPase for pumping protons out of the cell, hasty shutdown of CCMs, adjustment of fatty acid composition and diversion of excess photosynthates to generation of energy-rich compounds, such as triacylglycerols (Solovchenko and Khozin-Goldberg, 2013).

Microalgae also possess molecular machinery that allows them to distinguish between non-essential and essential heavy metals (Perales-Vela et al., 2006). This ability makes microalgae better suited for treatment of heavy metal polluted water, employing algal-based biotechnologies for waste-water remediation (Suresh Kumar et al., 2015). Indeed, microalgae take up and store toxic metals from the environment (Perales-Vela et al., 2006). For instance, microalgae can preferentially synthesize peptides capable to bind heavy metals. These peptides, as part of organometallic complexes, are additionally partitioned inside vacuoles to aid pertinent control of the cytoplasmic concentration of metal ions, thus nullifying their potential toxic effect (Cobbett and Goldsbrough, 2002). *C. reinhardtii* has been described as a model photosynthetic eukaryotic for investigating heavy metal tolerance or homeostasis due to molecular and genetic tools available for this species (Hanikenne, 2003). For example, phytochelatins (PC), low-molecular weight peptides, containing sulfur rich cysteine amino acid have been elicited as major intracellular cadmium (Cd) chelators in *C. reinhardtii* (Hu et al., 2001). In *Dunaliella tertiolecta*, PC, inducibly synthesized by zinc treatment, can function both in detoxifying heavy metals and alleviation of oxidative stress (Tsuji et al., 2002). Thioredoxins (TRXs) are also known to accord heavy metal detoxification in *Chlamydomonas* as exemplified by two *TRX* genes being differentially stimulated by Cd and mercury (Lemaire et al., 1999). As glutathione (GSH)-heavy metal adducts serve as substrates for PC synthase, an earlier study centered on two strains of *Scenedesmus acutus* (wild type and Cr^6+^ tolerant) reported the constitutive cysteine concentration higher in the Cr^6+^-tolerant strain (Torricelli et al., 2004). When the cells were subjected to Cd^2+^, the tolerant strain had increased levels of reduced GSH and class III metallothioneins (MtIII) as compared to the wild-type strain. Finally, proline (Pro) has also been reported to play a dominant role in alleviating environmental stress including heavy metal stress in plants, microorganisms and microalgae (Siripornadulsil et al., 2002).

Clearly, abiotic stress has the ability to reorient a holistic and sometimes specific microalgal metabolism as an efficient means for increasing the production of selected compounds. Keeping this in mind we highlight in the next section recent advances in microalgal engineering which, when combined with sophisticated physiological and biophysical approaches, may enhance microalgal stress resilience under disparate conditions.

### Examples of Stress-Resilience in Microalgae via Genetic Engineering

Despite most engineering strategies favoring increased production of high value compounds (e.g., antioxidant pigments and PUFAs) and biofuel-molecules (e.g., hydrogen and triacylgycerol), recent works have been driven to develop resilient strains with applications like CO_2_ sequestration and heavy metal biomitigation (**Table 1**). For example, a moth bean δ1-pyrroline-5-carboxylate synthetase (*P5CS*) gene has been expressed in *C. reinhardtii*, with transformants displaying 80% higher free-Pro levels, rapid growth at toxic Cd concentrations, and tremendous binding at four-fold higher Cd concentration compared to wild-type cells (Siripornadulsil et al., 2002). The results propose role of free-Pro as an antioxidant in Cd-stressed cells with resulting higher GSH levels facilitating enhanced phytochelatin synthesis and sequestration of Cd. Expeditious transgenesis has been reported using such microalgae to enhance heavy metal sensitivity and binding specificity for contaminated wastewaters and sediments (Rajamani et al., 2007). Much interest has also been diverted toward engineering small and large subunits of Rubisco (rbcS and rbcL) as targets for increasing net CO_2_ fixation (photosynthesis) and promoting CO_2_ sequestration through improvement of growth (Genkov et al., 2010; Whitney et al., 2011). In this regard, *Chlamydomonas* is a notable host for genetic manipulation, as changes can be made to both *rbcS* and *rbcL* genes. For instance, hybrid Rubiscos have been reported, by combining plant (*Arabidopsis*, spinach and sunflower) small (rbcS) subunits with algal large (rbcL) subunits via transformation of a *C. reinhardtii* mutant deficient in *rbcS* gene (Genkov et al., 2010). Although the hybrid enzymes show 3–11% increase in CO_2_/O_2_ affinity, they retain optimal V _max_ values and catalytically proficient Rubisco. The hybrid strains, however, lack chloroplast pyrenoids and display reduced photosynthesis. As asserted by Whitney et al. (2011), future research to engineer and test superior Rubiscos will considerably rely on algal model systems, particularly *Chlamydomonas.*

**Table 1:** Examples of green algal species aptly transformed to date together with methods applied and trait(s) enhanced for proficient metabolic or genetic engineering.

Algal species	Transfection method	Modification	Genes	Trait(s) engineered	Reference
*Chlamydomonas reinhardtii*	Glass beads	RNAi three target genes	*DGAT*	24 and 37% reduction in TAGs with two genes 34% increase in TAGs with one gene	Deng et al., 2012
	Glass beads	Nuclear overexpresion	*PSY*	2.6-fold increase in lutein	Couso et al., 2011
	–	Insertional mutagenesis	*CHT7*	Repressor of cellular quiescence under nitrogen-replete conditions	Tsai et al., 2014
	Biolistics	Chloroplast expression	*hemH* & *Iba*	Increase in hydrogen production	Wu et al., 2011
	Electroporation	Nuclear expression	*P5CS* (moth bean)	80% higher free-Pro levels, rapid growth under Cd toxicity, four-fold more binding to Cd	Siripornadulsil et al., 2002
	Glass beads	RNAi *LHC* gene family	20 genes encoding LHCI, LHCII, CP26 and CP29 down-regulated	Antenna-size reduction Increased competence for cell cultivation under high light intensity	Mussgnug et al., 2007
*Chlorella ellipsoidea*	Electroporation	Nuclear overexpression	Lipogenesis Transcription factors	52% increase in total lipids	Zhang et al., 2014
*Chorella minutissima*	Electroporation	Nuclear overexpression	Five genes encoding TAG enzymes	Twofold increase in TAGs with all five genes No change with individual gene	Hsieh et al., 2012
*Dunaliella salina*	Electroporation	RNAi	*PDS*	72% reduction of mRNA	Sun et al., 2008
*Haematococcus pluvialis*	Biolistics	Nuclear overexpression	*PDS*	26% increase in astaxanthin	Steinbrenner and Sandmann, 2006
*Nannochloropsis oceanica*	Electroporation	Nuclear overexpression	*Δ12-des*	Nitrogen-starvation induced increase deposition of polyunsaturated fatty acids (PUFAs) in TAG	Kaye et al., 2015

*CHT7, compromised hydrolysis of triacylglycerols 7; *Δ12-des*, Δ12-desaturase; *DGAT*, Acyl-CoA:diacylglycerol acyltransferase; *hemH*, ferrochelatase gene; *Iba*, leghemoglobin gene; *LHC*, light-harvesting antenna complexes; *P5CS*, δ1-pyrroline-5-carboxylate synthetase; *PDS*, phytoene desaturase; *PSY*, phytoene synthase; *TAG*, triacylglycerol.*

## Conclusion

Genetic manipulation of microalgae can provide feasible respite by meeting the immediate and long-term demands for food and fuel production on a sustainable basis. A clear step toward the realization of this goal is the development of microalgae-microbial fuel cells (mMFCs) that channelize the solar energy to electrical energy via algal metabolic pathways (Lee et al., 2015). Besides, genetic engineering of microalgae would supplement land plants and industrial biotechnology for value-added products, thereby saving land and air pollution. Already much is achieved through reinforced tools, including rapid sequencing of algal genomes, control of environmental stress conditions, as well as mining of new genes for enhancing lipid and bioactive productions, reducing photo-inhibition, and manipulation of Rubisco- and Calvin-cycle enzymes for metabolic engineering. The field of algal genetic engineering will require renewed attention both at basic and applied levels to envision a prosperous future.

## Author Contributions

FG and AK designed and conceptualized the idea and thereafter wrote different section or subsections in consultation with L-ST. All the authors read and finalized the version before submission.

## Conflict of Interest Statement

The authors declare that the research was conducted in the absence of any commercial or financial relationships that could be construed as a potential conflict of interest.

## Footnotes

1 www.ebi.ac.uk/biomodels-main

2 http://www.chlamycollection.org/

3 https://www.chlamylibrary.org/
